# The anthropometric determinants of peak expiratory flow rate among children in Dar Es Salaam, Tanzania

**DOI:** 10.1186/s12887-023-04520-1

**Published:** 2024-01-13

**Authors:** Willbroad Kyejo, Nancy Matillya, Neelam Ismail, Gloria Gachocha, Hajaj Salum, Rosebella Iseme, Mariam Noorani

**Affiliations:** 1 Department of Family Medicine, Aga Khan University, P.O. Box 38129, Dar Es Salaam, Tanzania; 2 Department of Pediatrics and Child health, Aga Khan University, P.O. Box 38129, Dar Es Salaam, Tanzania; 3 Department of Population Health, Aga Khan University, GPO, P.O. Box 30270-00100, Nairobi, Kenya

**Keywords:** Peak expiratory flow rate, Anthropometric measurements, Lung function, School children, Asthma, Dar es Salaam, Tanzania

## Abstract

**Background:**

Peak expiratory flow rate (PEFR) is an important tool for assessing lung function, which can be affected by environmental and physical factors such as altitude, nutrition, genetics, age, height, and weight. Conducting a study to assess the correlation between peak expiratory flow rate and anthropometric measurements in Tanzanian schoolchildren is crucial to derive a population-specific prediction formula and further simplify respiratory health assessment.

**Methods:**

This cross-sectional study was conducted in a single center private primary and secondary school in Dar es Salaam, Tanzania using data from an asthma screening camp. Variables of interest were height, weight, Body Mass Index (BMI) and PEFR. Independent t-test was performed to identify any differences in mean flow rate values between different ethnicities and genders. Correlation coefficients (r) were used to observe the relationship between PEFR and anthropometric measurements. A prediction equation by gender was generated using linear regression analysis. Statistical significance was set at the 5% level. All statistical data was analyzed using SPSS version 25.0.

**Results:**

The study involved 260 participants with a mean age of 9.5 years. Males were 51.2% and 65% of participants were of Asian ethnicity. PEFR was not observed to differ across the different ethnic groups and genders. Height was found to have the strongest correlation coefficient of 0.745, while BMI had the weakest correlation coefficient of 0.366. The strongest correlation was found with height for females (*r* = 0.787), while the weakest was with body mass index for boys (*r* = 0.203). The derived prediction equation for males was **PEFR = 279.169 (Height of Student in meters) —134.12**, while the predictive equation for females was **PEFR = 318.32 (Height of Student in meters) —195.69**.

**Conclusion:**

This study found a strong correlation between PEFR and anthropometric characteristics in school children from Dar es Salaam, Tanzania. A prediction equation by gender for PEFR was developed based on anthropometric characteristics. This equation may be applied in population-based studies or situations where peak flow meters are not readily available. Further research is needed to explore how well this prediction formula performs in other Tanzanian settings and to determine other factors that may affect lung function in this population.

## Introduction

Lung function tests have evolved from tools for physiological study to clinical investigations in assessing respiratory status. They have become a part of routine health examination in respiratory, occupational, sports medicine and public health screening [1]. Tests have been designated to indicate the extent of narrowing of airways, which can lead to breathing issues, decreased oxygen consumption, and an increased risk of respiratory infections. A simple but important test used to measure lung function is the Pulmonary function test (PFT) [2]. 

The PFT is a basic tool for assessing lung dysfunction, disease, and prognosis of treatment [1]. Peak expiratory flow rate (PEFR) is a PFT which measures the maximum speed of airflow attained by a forceful, complete expiration after complete inhalation [2, 3]. It was introduced by Hadron in 1942 and accepted in 1949 as an index in spirometry [4]. Notably, although PFT is acknowledged to provide valuable information it is not without its limitations. In this regard, it can be physically demanding for patients and dependent on patient effort, understanding of and cooperation with instructions as well as variations in technique. Intra individual variation in pulmonary function can also be attributed to circadian rhythm, host factors, size, age, past and present health, and geographic factors [4, 5]. Consequently, each country and region should have its own PEFR standard reference values [6]. 

Gender differences in airway behavior and clinical manifestations of airway disease have also been reported. The latter are attributed to biological and socio-cultural factors, such as body size, sex hormones, sex hormone receptors, and intracellular signaling pathways [7, 8]. Ethnicity has also been shown to have an impact on lung function variation, with white populations having higher Forced Vital Capacity and Forced Expiratory Volume in 1 s (FEV1), and black Americans having smaller lung volumes [3, 8]. Additionally, increased weight decreases lung volume and capacities, this is due to increasing resistance to outflow of air through the airways. PEFR is positively correlated with height and inversely correlated with weight. Obesity is linked to decreased PFTs due to its restrictive effect on the lung and chest wall [7, 9, 10]. Notably, studies have reported a significant increase in PEFR with age in children, with boys having higher values than girls. The relationship between age and PEFR in children is complex and may be influenced by sex, ethnicity, physical activity levels, and the method used to measure PEFR [8, 11, 12]. 

The present study was designed to measure the PEFR of boys and girls attending a single private primary and secondary school in Tanzania and examine how it correlates with anthropometric measurements. The data was then used to derive a prediction formula which can potentially be used in this population. The developed PEFR prediction formula based on anthropometric measurements in Tanzanian school children serves as a crucial tool for identifying potential lung dysfunction and respiratory complications, facilitating simpler respiratory assessment and timely interventions.

## Materials and methods

The analytical cross-sectional study was conducted at Aga Khan Mzizima School, encompassing both primary and secondary school children in Dar es Salaam, Tanzania. The school opened in 1993, and its private setting accommodates students of various ethnicities and backgrounds. It predominantly comprises students from families of middle to upper socio-economic status. The study was carried out during a designated school asthma screening camp held from June 7th to June 10th, 2022. This camp served as an opportune platform to gather data pertaining to respiratory health among students.

The study was approved by the Aga Khan University, East Africa Ethics Review Committee (approval no. AKU/2023/03/fb/05/03), aimed primarily to observe the correlation of peak expiratory flow rate with anthropometric determinants in a population of children attending a single private primary and secondary school in Tanzania.

All students from primary and secondary school who consented to participate in the screening camp were recruited while excluding students who were known to have a respiratory condition such as asthma or had symptoms consistent with asthma.

The sample size for estimating correlation between variables was used.

n = [(Zα/2 + Zβ) / C(r)]2 + 3 where: n is the sample size needed ,Zα/2 is the critical value of the standard normal distribution at the desired level of significance for a 2-sided test (i.e., 1.96 for α = 0.05), Zβ is the critical value of the standard normal distribution at the desired power level (1- β) (i.e., 1.282 for β = 0.1) ,Cr is Fisher’s transformation and is equal to 0.5 × ln[(1 + r)/(1-r)], r is the expected correlation coefficient (0.7) [11].

Cr = 0.5 × ln[(1 + r)/(1-r)] = 0.5 × ln [(1 + 0.7)/ (1-0.7)] = 0.5 × ln [1.7/0.3] = 0.5 × ln 5.67 = 0.5 × 0.75 = 0.3769.

*n* = 77, Inflate of 10% was added to the sample size accounting for spoiled records or missing data (*n* + 8). Therefore, the minimum sample size required for the study was 85 students; All eligible students attending the asthma screening camp were enrolled in the study using a census sampling approach, encompassing those who met the eligibility criteria, provided consent, and were affiliated with the school for convenience.This inclusive approach not only exceeded the minimum sample size but also significantly enhanced the statistical power of the analysis. By incorporating data from all eligible participants, the study aimed to maximize its statistical strength, ensuring a robust investigation into the relationship between anthropometric variables and PEFR.

Data collection was overseen by medical professionals from Aga Khan Hospital during a dedicated school asthma screening camp. Prior to the screening, parents or guardians received screening questionnaires and consent forms. Only children with parental consent participated in the screening, subsequently being enrolled in the study.

Throughout the screening process, essential variables like age, height, and weight were documented for each participant. The study focused on PEFR, a pivotal measure of lung function critical in diagnosing and managing respiratory conditions, notably asthma. PEFR assessments were conducted using a ‘breath-o-meter’ peak flow meter provided by Chemical Industrial & Pharmaceutical Laboratories Ltd company. While seated, participants underwent three separate PEFR readings. Notably, the highest recorded value among these three measurements was considered as the participant’s PEFR. Capturing the maximum expiratory flow potential was the rationale behind selecting the highest among the three PEFR readings. This approach aimed to offer a more accurate representation of the participants’ respiratory capabilities during the assessment, considering PEFR as a key outcome variable.

Independent t-test was used to examine differences in PEFR between Asian and African children and between boys and girls. Correlation coefficients were used to assess the relationship between PEFR and height, weight, age, and BMI. A prediction equation was generated using linear regression analysis. Statistical significance was set at 5%. All statistical data was analyzed using SPSS version 25.0.

## Results

**Fig. 1:**
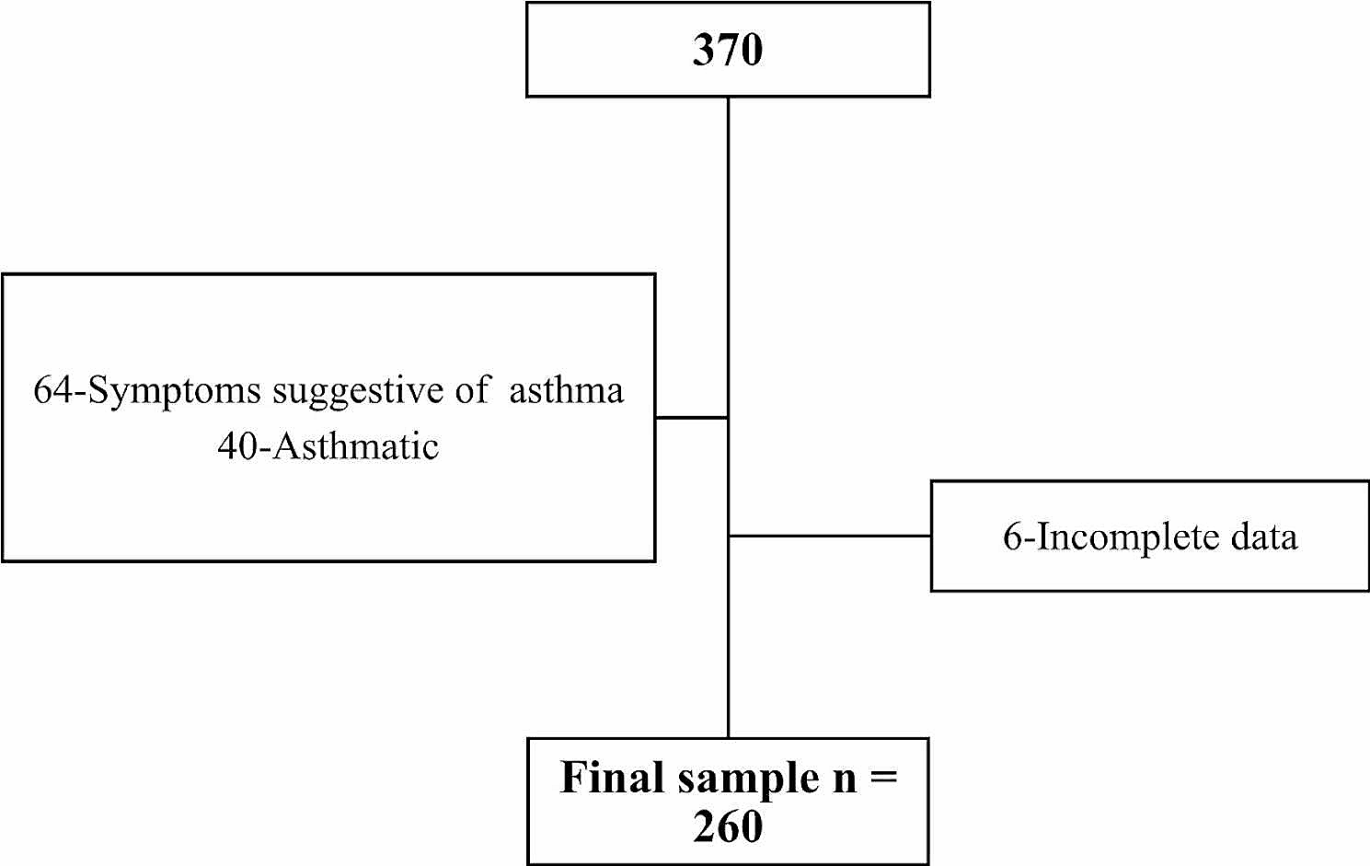
Participant recruitment flow

A total of 370 school students were recruited between April to May 2022. Of these participants, 110 were excluded, of whom 6 had incomplete data, 40 had a known diagnosis of asthma and 64 had symptoms consistent with asthma. A total of 260 participants were enrolled in the study, as shown in the flow chart (Fig. 1).

Of the participants, 133 (51.15%) were male, 169 (65%) were of Asian ethicinity and the mean age was 9.47 years (SD 1.78) with an age ranging from 6 to 17 years old. The mean was PEFR 238.32 L/min (SD 48.50). The sociodemographic and clinical parameters are summarized in Table 1.

**Table 1:** Sociodemographic and clinical parameters of the study population

Variable	Frequency		
**Age (Mean; SD)**	9.47 (1.78)		
**Sex (n; %)**			
Male	133 (51.15)		
Female	127 (48.85)		
**Ethnicity (n; %)**			
Asian	169(65.00)		
African	91 (35.00)		
**Height meters (mean; SD)**	1.35 (0.12)		
**Weight kg (mean; SD)**	35.51 (13.02)		
**BMI continuous (kg/m** ^**2**^ **) (mean; SD)**	19.19 (4.85)		
BMI categorical* (n; %)			
Underweight:	22 (8)		
Healthy:	113 (43)		
Overweight:	43 (17)		
Obese:	82 (32)		
**PEFR (mean; SD)**	238.32 (48.50)		

*BMI categories determined based on WHO BMI charts for age and sex

An Independent Sample t-test was conducted to compare PEFR between male and female pupils as well as across the 2 main ethnic groups, i.e., Asian, and African. The results of the two-tailed test indicated that there was no significant difference in PEFR between males (mean = 238.46, SD = 45.00) and females (mean = 238.18, SD = 52.09); t (258) = 0.96, p-value = 0.96.

Similarly, no statistically apparent difference in PEFR was observed between Asian pupils (mean = 234.02, SD = 48.805) and African pupils (mean = 246.32, SD = 47.145), t-(258) = 1.96, p-value = 0.051.

Table 2 shows the Pearson correlation indicating the strength of the relationship between anthropometric parameters and PEFR, stratified between female and male participants. The results suggest that age, height, weight, and BMI were all statistically significantly correlated with PEFR. Notably the strongest correlation was found between height and PEFR for females and the weakest correlation was observed between BMI and PEFR for males. Interestingly all anthropometric measures were positively related with PEFR across both genders, i.e., as age, height, weight, and BMI increase so too does PEFR.

**Table 2:** Pearson correlation of anthropometrical parameters with PEFR in the entire study group, female, and male subjects

Variable	Total		Female		Male	
	Correlation coefficient (r)	P value	Correlation coefficient (r)	P value	Correlation coefficient (r)	P value
Age	0.697	0.000	0.745	0.000	0.648	0.000
Height	0.745	0.000	0.787	0.000	0.760	0.000
Weight	0.627	0.000	0.676	0.000	0.569	0.000
BMI	0.366	0.000	0.479	0.000	0.203	0.019

A linear regression analysis was conducted separately for female and male students to explore the relationship between PEFR and height, using height as the independent variable and PEFR as the dependent variable. Even when considering potential additional variables like age or weight, the analysis revealed that the model’s predictive strength remained robust solely with height as a predictor. This decision was guided by the strong correlation observed between height and PEFR within the Tanzanian child population, rendering the inclusion of other variables unnecessary for enhancing predictive accuracy.

Table 3 presents results of a regression analysis showing that an increase in height (in meters) is significantly and positively associated with a dependent variable (PEFR), across both female and male children.

**Table 3:** Results of the linear regression analysis of PEFR and height (in meters)

Variable	Unstandardized Beta coefficients	Standardized Beta Coefficients	p value	Standard error	95% CI
Height (**Male**)	279.170	0.741	0.00	22.14	235.41– 322.93
Height (**Female**)	318.320	0.761	0.00	24.24	270.46–366.17

**Fig. 2:**
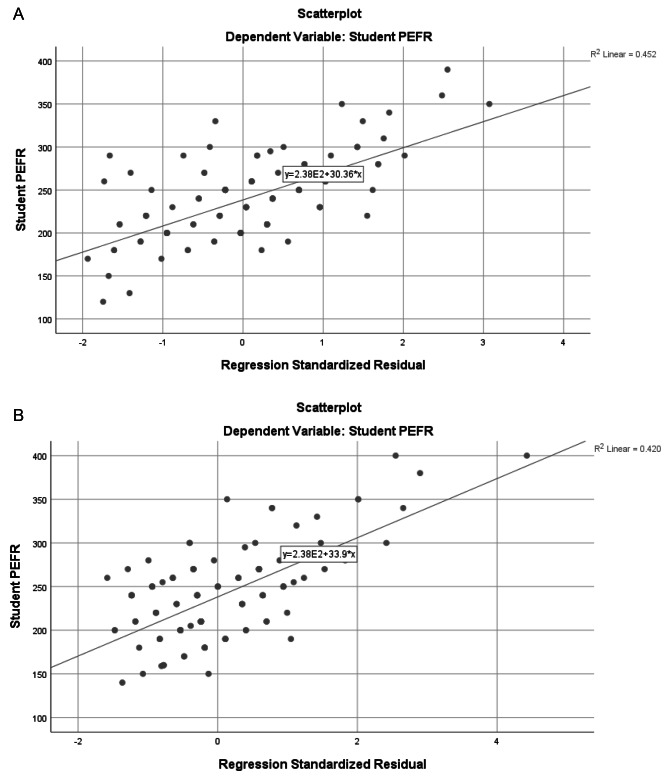
**a**: Linear regression between male student PEFR and standardized residual of the Height. The regression equation derived from the linear regression above (Fig. 2), used to predict male PEFR based on height, is: **PEFR = 279.169 (Height of Student in m) —134.12. b**: Linear regression between female student PEFR and standardized residual of the Height. The regression equation derived from the linear regression above (Fig. 2), used to predict female PEFR based on height, is as outlined below: **PEFR = 318.32 (Height of Student in m) —195.69**

Figure 2a and b below show the scatterplots of the linear regression model for male and female students respectively.

## Discussion

This study provides valuable insights into the correlation between anthropometric variables (age, height, weight and BMI) and PEFR in school children from Dar es Salaam, Tanzania. The results show that age, height, weight, and BMI are significantly correlated with PEFR, with height demonstrating the highest correlation coefficient (*r* = 0.745, *p* < 0.001).

The strong correlation between height and PEFR in the Tanzanian child population aligns with previous research conducted in diverse populations. Studies conducted in various countries and age groups have consistently demonstrated a positive relationship between height and lung function parameters, including PEFR [13, 14]. This steadfast consistency across distinct demographic cohorts fortifies the applicability of the height-PEFR correlation and validates its reliability as a pragmatic tool for evaluating respiratory well-being. As a consequence, the adoption of height as a solitary predictor for estimating PEFR exhibits the potential to rationalize and simplify assessment protocols, particularly in settings characterized by limited resources, where access to specialized equipment might be constrained [13, 14]. .

In this study, we observed a correlation coefficient of 0.672 between weight and PEFR, indicating a moderate correlation between these two variables. This finding suggests that there is a noticeable relationship between weight and lung function, where higher weight is associated with changes in PEFR. However, these results appear to be contrary to previous studies that have shown either no correlation or a weaker correlation between weight and lung function. This discrepancy may be attributed to the presence of adipose fat around the chest, which could limit lung compliance and volume, thereby potentially affecting the expected correlation [1, 15]. However, research is warranted to delve into the underlying mechanisms and potential contributing factors that may be driving this correlation. Specifically, investigating these aspects in the context of children’s lung function is of particular importance, as it can provide insights into the unique dynamics of respiratory development and potential health implications.

Interestingly, the study reveals a weak correlation of 0.366 between BMI and PEFR, particularly among boys( *r* = 0.209). This finding suggests that it may be more informative to consider individual variables such as height rather than relying on a combined measure like BMI when assessing the relationship with lung function. This finding is in line with some previous studies but contradicts others that reported a significant association between BMI and lung function in children [11]. The weak correlation observed in this study could be attributed to the high prevalence of overweight and obesity among participants, where the impact of excess weight on lung function becomes less discernible or diluted. Other factors such as body composition, fat distribution, or specific physiological mechanisms might also contribute to this weak correlation.

Furthermore, it is worth noting that the overall correlation coefficients reported in our study were lower compared to some previous studies. For instance, a study conducted in Sri Lanka among healthy school children found very high correlations between PEFR and height, weight, and BMI (*r* = 0.89, 0.86, and 0.891, respectively) [7]. Similarly, a study conducted in Nigeria among school children reported strong correlations between PEFR and height, weight (*r* = 0.77 and 0.76 respectively) [11]. The relatively lower correlation coefficients in our study could be attributed to the smaller sample size and variations in the characteristics of the study population, such as age range, ethnicity, and environmental factors [7]. 

The congruence between the study findings and prior research conducted by Seema S. et al., which also identified no statistically significant disparity in PEFR between males and females, underscores the consistency in these observations [16]. . This result shows there is no significant variation in PEFR between males and females, indicating that gender does not play a substantial role in influencing this respiratory measure. It provides valuable insights to healthcare professionals and helps them comprehend the impact of gender on PEFR within the confines of this study.

However, it is essential to remain cautious and consider the potential influence of gender in other populations or for different health conditions. On the other hand, concerning the association between ethnicity and PEFR, our study did not find a statistically significant difference between Asian and African populations, which aligns with the findings of Whittaker A et al. 2005 [15, 17]. Nevertheless, it is essential to note that some studies have reported significant variations in PEFR across different ethnic groups, as indicated by Udupihille M et al. 1994 [15].Several factors could contribute to these variations, including genetic factors influencing lung function, disparities in environmental exposures such as air pollution and indoor air quality, socioeconomic status, lifestyle habits, cultural practices, and healthcare disparities.

Based on the derived formula from the study, which establishes a significant relationship between PEFR and height, a prediction model has been developed. The formula, predictive equation for male **PEFR = 279.169 (Height of Student in m) —134.12** while predictive equation for female is **PEFR = 318.32 (Height of Student in m) —195.69**, the formula can be utilized to predict the PEFR of male and female individuals based on their height. The coefficient values indicate that height holds the strongest positive relationship with PEFR.

Comparing the derived formula from this study to existing formulas used in Nigeria, USA, India, and Europe reveals intriguing findings. Our formula, PEFR = 293.04 (Height of Student in meters) —157.362, shares a notable similarity with the formula derived in Nigeria, PEFR = 345(Height) —222. Both formulas primarily rely on height as the predictor for PEFR, and interestingly, in both of these studies, height alone yields a high R² compared to when other variables are combined. The results obtained from our formula and the Nigerian formula exhibit a small difference in standard deviation, approximately +/- 10. This suggests that our formula yields similar results with a slight variation [11]. 

However, when comparing our formula to those used in the USA (PEFR(l/min) = [Height,cm-100)*5] + 100 (22)) and India (PEFR(l/min) = (19.964 * age in years) —(0.0988 * height in cm) + 32.455 (23)), slight differences in standard deviation, around +/- 5, are observed when compared to our formula. Remarkably, even in the USA formula, where height alone is used in linear regression, it yields a higher R² compared to when combined with other variables. Intriguingly, in the Indian study, the author employed both age and height since the R² remains consistent whether combined or used as independent predictors. These formulas employed in children populations incorporate additional predictors such as age. Moreover, these variations may be attributed to differences in ethnicity and genetic factors, which can influence respiratory function [5]. 

The successful development of this prediction model as well as variability in results when comparing studies undertaken in different populations strongly advocates the importance of establishing a locally derived reference standard for PEFR in the Tanzanian child population and the broader East African region. This model’s effectiveness in estimating PEFR using only height, which is easily measurable, highlights its potential to significantly enhance healthcare practices in resource-limited settings. By implementing this formula, healthcare professionals in these regions can accurately estimate a child’s PEFR without the need for expensive and inaccessible equipment. This represents a crucial advancement, as traditional methods that rely on specialized devices can be cost-prohibitive and challenging to obtain, particularly in low-income or remote areas.

In conclusion, this study provides valuable insights into the relationship between anthropometric variables and PEFR in school children from Dar es Salaam, Tanzania. The findings contribute to the existing body of knowledge and highlight the importance of locally derived reference standards for accurate PEFR estimation in this population. Further research is needed to explore additional factors and validate the prediction model in larger and more diverse populations.

### Limitations

An important limitation lies in the study’s focus on school children from a sole institution in Dar es Salaam, Tanzania. As such, the findings might not be broadly representative of other populations, including those in rural areas or children from diverse socioeconomic backgrounds. This limitation impacts the generalizability of the results beyond the specific urban school setting under investigation.

## Conclusion

The results of this study suggest that there is a strong correlation between peak expiratory flow rate (PEFR) and age, height, and weight, and a weaker correlation with body mass index (BMI) in school children from Dar es Salaam, Tanzania. These findings highlight the importance of anthropometric characteristics in predicting lung function in this population. The study also developed a predictive equation for PEFR in this population based on their anthropometric characteristics, which could be useful in clinical practice and research. Further research is needed to explore other factors that may affect lung function in this population, including environmental factors, lifestyle behaviors, and socioeconomic status.

## Declarations

### Data sharing statement

The datasets used and/or analyzed during the current study are available from the corresponding author on reasonable request.
